# Baseline and Cumulative Blood Pressure in Predicting the Occurrence of Cardiovascular Events

**DOI:** 10.3389/fcvm.2021.735679

**Published:** 2021-09-21

**Authors:** Yingting Zuo, Deqiang Zheng, Shuohua Chen, Xinghua Yang, Yuxiang Yan, Fen Liu, Xue Tian, Meiping Wang, Xin Su, Jing Wen, Qi Zhai, Yibo Zhang, Herbert Y. Gaisano, Shouling Wu, Anxin Wang, Yan He

**Affiliations:** ^1^Department of Epidemiology and Health Statistics, School of Public Health, Capital Medical University, Beijing, China; ^2^Beijing Municipal Key Laboratory of Clinical Epidemiology, Beijing, China; ^3^Department of Cardiology, Kailuan Hospital, North China University of Science and Technology, Tangshan, China; ^4^Departments of Medicine and Physiology, University of Toronto, Toronto, ON, Canada; ^5^Department of Neurology, Beijing Tiantan Hospital, Capital Medical University, Beijing, China; ^6^China National Clinical Research Center for Neurological Diseases, Beijing Tiantan Hospital, Capital Medical University, Beijing, China

**Keywords:** hypertenion, baseline blood pressure, exposure, time-weighted cumulative blood pressure, cardiovasclar disease

## Abstract

**Background:** Both baseline blood pressure (BP) and cumulative BP have been used to estimate cardiovascular event (CVE) risk of higher BP, but which one is more reliable for recommendation to routine clinical practice is unclear.

**Methods:** In this prospective study, conducted in the Kailuan community of Tanshan City, China, a total of 95,702 participants free of CVEs at baseline (2006–2007) were included and followed up until 2017. Time-weighted cumulative BP that expresses the extent of cumulative BP exposure is defined as the sum of the mean of two consecutive systolic or diastolic BP times the interval between the two determinations, then normalized by the total follow-up duration. Incident CVEs during 2006–2017 were confirmed by review of medical records. We performed a competing risk regression analysis to assess CVE risk of the different durations of higher BP exposure. ROC analysis was performed to assess the predictive value of higher BP on CVE occurrence.

**Results:** We found that when the risk of higher BP on CVE occurrence was estimated based on time-weighted cumulative BP, the hazard ratios (HRs) increased with the increase in duration of higher BP exposure in each of the four BP groups: <120/<80, 120–129/<80, 130–139/80–89, and ≥140/≥90 mmHg; this time trend also occurred across the four different BP groups, with the higher BP group exhibiting CVE risk earlier during the follow-up. These results were confirmed by the same analysis performed on participants without baseline hypertension. However, such reasonable time trends did not occur when a single baseline BP was used as the primary estimation. We also demonstrated that the predictive values of baseline systolic and diastolic BP that predict CVE occurrence were only 0.6–3.2 and 0.2–3.1% lower, respectively, than those of cumulative BP combined with baseline BP during follow-up.

**Conclusions:** Baseline BP remains a useful indicator for predicting future occurrence of CVEs. Nevertheless, time-weighted cumulative BP could more reliably estimate the CVE risk of higher BP exposure than baseline BP.

## Introduction

Hypertension is highly prevalent in the adult population, affecting about 1.13 billion adults worldwide, of which 226 million are in China (1). A previous study reported that ~25% of cardiovascular events (CVEs) were attributable to hypertension (2). Therefore, a precise assessment of the influence of high blood pressure (BP) exposure on the occurrence of CVEs is critically important to gain a more complete understanding of CVE risk of hypertension.

For reasons of accessibility, the effect of a higher BP on CVEs has been frequently estimated based on baseline BP measurement (3–8). The presumption of this common practice is that future BP is better predicted by the current BP, but this presumption is actually misleading since BP changes particularly with the increase in age (9–11). More recently, the cumulative BP determination was developed, which includes several BP measurements that take into consideration of both how high the BP levels have been and the duration of the high BP exposure before risk estimation. Several studies have simultaneously used the cumulative BP and baseline BP determinations to assess the effect of high BP on CVE occurrence, which showed that the association of the cumulative BP determination with CVE was stronger than the baseline BP determination (12–15). Although cumulative BP seems to be superior than baseline BP in estimating CVE risk of high BP theoretically, no population studies have yet to verify this presumption. Thus, overall, it remains unclear whether cumulative BP vs. baseline BP determination is more reliable for recommendation to routine clinical practice.

Currently, baseline BP determination remains commonly used to predict the occurrence of CVEs. Two studies compared a single BP determination vs. cumulative BP determination in its predictive ability on the occurrence of cardiovascular disease (CVD), which found that cumulative BP determination had an improvement in disease risk prediction models for the improvement of net reclassification index (NRI) or C-statistics (15, 16). However, these studies did not demonstrate the individual predictive values (AUC) of cumulative BP and a single measurement of BP determinations; therefore, the absolute deviation between the two remains equivocal. This raises the unresolved issue that baseline BP determination could still be used as an indicator to predict the occurrence of CVEs.

To address the abovementioned issue, we have leveraged the Kailuan Study, a large prospective cohort study. We first aimed to compare the performance of baseline BP vs. cumulative BP determinations in estimating CVE risk of different durations of high BP measured during follow-up; and secondly, we compared the predictive value of baseline BP vs. cumulative BP determinations in predicting CVEs during each follow-up.

## Methods

### Study Design and Participants

The data of this prospective study were derived from the Kailuan Study, which was a prospective cohort study that was conducted in the Kailuan community in Tanshan City, China (17). The participants in the Kailuan Study were employees and retirees of the Kailuan Group Company, which is the largest company of the coal mining industry in Tangshan. In brief, a total of 101,510 individuals (81,110 men and 20,400 women, aged 18–98 years) completed the first survey conducted between June 2006 and October 2007. We performed re-examinations at 2-year intervals up to the end of the last follow-up on December 31, 2017. The detailed design of the Kailuan Study has been described previously (18, 19).

In the present study, we excluded the following: 3,732 participants that had a history of cardiovascular disease, 1,159 participants with missing information on BP measurements, and 917 participants with hypotension at baseline. Therefore, a total of 95,702 participants were eligible to be included, of which 88,396 completed the 11 years of follow-up (Supplementary Figure 1). This study was approved by the Ethics Committees of Kailuan General Hospital and Beijing Tiantan Hospital and adhered to the principles of the Helsinki Declaration. All participants signed an informed consent.

### Data Collection

In the Kailuan Study, all participants completed a questionnaire, which included information on age, sex, smoking, alcohol intake, physical activity, and medical history. Current smoking was defined as smoking for at least in the last year. Current alcohol consumption was defined as the average daily consumption of a strong spirit (alcohol content >50%) of 100 ml or more for at least in the previous year. Active physical activity was categorized as very active (≥80 min/week of moderate to vigorous intensity), moderately active (<80 min/week of moderate to vigorous intensity), and inactive (no exercise at all). Antihypertensive medication was defined as any self-reported use of antihypertensive drugs.

Anthropometric measurements included height and weight, and body mass index was calculated as weight in kilograms divided by height in meters squared. BP was measured with a manual sphygmomanometer in the period between 2006 and 2014 and with an electronic blood pressure meter (HEM-8102A; Omron Limited, Dalian, China) from 2014 onwards (19). Three readings of systolic blood pressure (SBP) and diastolic blood pressure (DBP) were taken at a 5-min interval after the participants had rested in a seated position for at least 5 min. The reading values were rounded to the nearest full figure. The average value of the three BP measures was used for further analysis.

Blood samples taken from the antecubital vein in the morning after an overnight fast (8–12 h) were assayed with an automatic analyzer (Hitachi 747; Hitachi, Tokyo, Japan) for fasting plasma glucose, total cholesterol, triglycerides, high-density lipoprotein cholesterol and low-density lipoprotein cholesterol levels, and creatinine (20). The estimated glomerular filtration rate was calculated by the Chronic Kidney Disease Epidemiology Collaboration equation (21).

The diagnostic criteria for hypertension are as follows: a SBP ≥140 mmHg or DBP ≥90 mmHg, a diagnosis of hypertension by a previous physician, or the use of antihypertensive drugs (22). Hypotension was defined as SBP ≤90 mmHg or DBP ≤60 mmHg. Variability of SBP and DBP was estimated as the standard deviation (SD) of the measures during the whole follow-up periods. Diabetes was defined as a self-reported history of a specialist-made diagnosis of diabetes mellitus, the intake of hypoglycemic drugs, or a fasting plasma glucose ≥7.0 mmol/L (23).

### Calculation of Time-Weighted Cumulative BP

To precisely determine the extent of BP exposure of an individual, a time-weighted cumulative BP determination using all available BP measurements from baseline to the end of this study or before any incident CVE was calculated (13). The time-weighted cumulative BP determination was defined as the sum of the mean of each two consecutive SBP or DBP times its corresponding interval and then normalized by the follow-up time of the individual. For example, if a participant was enrolled in 2006 and followed up in 2008, 2010, and 2012, then the time-weighted cumulative BP in 2012 was calculated as [(BP_2006_ + BP_2008_)/2 ^*^ 2 + (BP_2008_ + BP_2010_)/2 ^*^ 2 + (BP_2010_ + BP_2012_)/2 ^*^ 2]/6. Baseline and cumulative BP determinations were categorized into four mutually exclusive groups: (1) <120/<80 mmHg: SBP <120 mmHg and DBP <80 mmHg, (2) 120–129/<80 mmHg: SBP 120–129 mmHg and DBP <80 mmHg, (3) 130–139/80–89 mmHg: SBP 130–139 mmHg or DBP 80–89 mmHg, and (4) ≥140/≥90 mmHg: SBP ≥140 mmHg or DBP ≥90 mmHg.

### Assessment of Incident CVEs

The participants of the present study were followed up from the baseline examination in 2006 up to the date of the occurrence of CVEs, or up to 2017 that is the very end of the follow-up period for this study, whichever came first. A CVE is the main outcome, which include stroke, myocardial infarction, and cardiovascular death (24). Stroke was diagnosed according to the World Health Organization criteria on the basis of clinical symptoms, images obtained by computed tomography or magnetic resonance imaging, and other diagnostic reports (25). Myocardial infarction was diagnosed according to the criteria of the World Health Organization based on clinical symptoms, electrocardiogram changes and cardiac enzyme levels, and various symptoms of chest pain (26). Cardiovascular death was defined as death from cardiovascular disease according to the tenth version of the International Classification of Disease (ICD-10). In this study, the outcomes information was obtained directly from the regular re-examinations conducted at 2-year intervals or from the hospital discharge summaries and medical records from the Hospital Discharge Register and the Municipal Social Insurance.

### Statistical Analyses

Data were expressed as frequency and percentage, median with interquartile range, or mean ± standard deviation. Tests of differences in the characteristics across the categories for baseline BP determination were performed using the analysis of variance or the Kruskal-Wallis test for continuous variables according to distribution and the chi-square test for categorical variables. The incidence of CVEs was expressed as incidence density (per 1,000 person-years).

Univariate survival analysis was performed by the Kaplan–Meier method and log-rank test. To eliminate the bias from the competing risk of death, a Fine and Gray competing risk regression was constructed to examine the association of BP and the incidence of CVEs, whereas non-cardiovascular death was treated as a competing risk event (27). To assess the association of BP and the incident CVEs, we fitted three regression models. Model 1 was adjusted for age, sex, body mass index, smoking, alcohol consumption, physical activity, and SBP and DBP at baseline. Model 2 was adjusted for all the covariates in model 1 plus triglycerides, low-density lipoprotein cholesterol, high-density lipoprotein cholesterol, fasting plasma glucose, and estimated glomerular filtration rate at baseline. Model 3 was further adjusted for history of hypertension and antihypertensive drug intake at baseline and the variation of SBP and DBP during follow-up based on model 2.

Restricted cubic spline regression was established to examine the optimal range of BP by adjusted hazard ratios (HRs) and 95% confidence intervals (CIs), with 5 knots located at the 5th, 25th, 50th, 75th, and 95th percentiles of SBP or DBP (28). First, through the smooth curve and Cox regression model, we could find the point with the lowest risk of CVEs. Second, we used this lowest point as the reference to determine a non-significantly optimal range. The predictive values of baseline BP and time-weighted cumulative BP determinations for CVE occurrence were evaluated by the AUC, which was calculated from the receiver operating characteristic (ROC) curve analyses.

All analyses were conducted using SAS version 9.4 (SAS Institute Inc., Cary, NC, USA) or R version 3.6.1 (R Foundation for Statistical Computing, Vienna, Austria). All reported *P*-values were based on a two-sided test of significance, and *P* < 0.05 was deemed statistically significant.

## Results

### Baseline Characteristics of the Participants

The median age of the participants in the study was 51.6 years (IQR 43.5–58.8) and males accounted for 79.7% (*N* = 76,310). Table 1 summarizes the baseline characteristics of the participants along the different baseline BP categories. Participants with a BP of ≥140/≥90 mmHg were older and had higher body mass index, fasting plasma glucose, low-density lipoprotein cholesterol, triglycerides, and total cholesterol and lower estimated glomerular filtration rate. The proportion of participants with diabetes and dyslipidemia was highest in the group with BP ≥140/≥90 mmHg.

**Table 1:** Characteristics of the participants according to the baseline BP categories.

**Baseline characteristic**	** <120/<80 mmHg**	**120–129/<80 mmHg**	**130–139/80–89 mmHg**	**≥140/≥90 mmHg**	***P*** **-value**
No. (%)	18,999 (19.8)	5,781 (6.0)	43,847 (45.8)	27,075 (28.3)	
Age, years	46.4 (36.9–53.8)	51.7 (43.3–59.3)	51.7 (43.7–58.9)	54.3 (48.3–61.8)	<0.01
Male sex, *n* (%)	12,688 (66.8)	4,501 (77.9)	35,868 (81.8)	23,253 (85.9)	<0.01
Current smoker, *n* (%)	6,623 (35.4)	2,258 (40.1)	14,643 (34.4)	8,367 (31.8)	<0.01
Current alcohol, *n* (%)	7,479 (40.0)	2,405 (42.7)	16,014 (37.6)	8,998 (34.2)	<0.01
Physical activity, *n* (%)					
Inactive	1,834 (9.9)	506 (9.0)	3,721 (8.8)	2,008 (7.7)	<0.01
Moderately active	14,303 (77.4)	4,137 (74.0)	32,041 (76.1)	19,761 (75.7)	
Very active	2,333 (12.6)	950 (17.0)	6,328 (15.0)	4,321 (16.6)	
BMI, kg/m^2^	23.4 (21.3–25.6)	24.3 (22.1–26.6)	24.9 (22.8–27.2)	26.0 (23.8–28.3)	<0.01
SBP, mmHg	110.0 (100.7–111.0)	120.7 (120.0–128.0)	130.0 (120.0–134.0)	150.0 (140.0–160.0)	<0.01
DBP, mmHg	70.0 (69.3–73.3)	75.0 (70.0–79.3)	80.0 (80.0–85.0)	96.0 (90.0–100.0)	<0.01
FPG, mmol/L	5.0 (4.6–5.4)	5.1 (4.6–5.6)	5.1 (4.7–5.7)	5.2 (4.8–6.0)	<0.01
LDL-C, mmol/L	2.2 (1.8–2.7)	2.3 (1.8–2.8)	2.4 (1.8–2.8)	2.4 (1.9–2.9)	<0.01
HDL-C, mmol/L	1.5 (1.3–1.7)	1.5 (1.3–1.8)	1.5 (1.3–1.8)	1.5 (1.3–1.8)	<0.01
TG, mmol/L	1.0 (0.7–1.6)	1.2 (0.8–1.7)	1.3 (0.9–2.0)	1.4 (1.0–2.2)	<0.01
TC, mmol/L	4.8 (4.1–5.4)	4.9 (4.3–5.6)	4.9 (4.3–5.6)	5.0 (4.4–5.7)	<0.01
eGFR (ml/min/1.73 m^2^)	86.3 (73.8–99.7)	82.6 (70.4–95.4)	81.5 (68.0–96.1)	75.7 (63.3–90.8)	<0.01
History of hypertension, *n* (%)	398 (2.1)	346 (6.0)	3,947 (9.0)	6,289 (23.2)	<0.01
Diabetes mellitus, *n* (%)	874 (4.6)	469 (8.1)	3,878 (8.8)	3,437 (12.7)	<0.01
Dyslipidemia, *n* (%)	4,906 (25.8)	1,850 (32.0)	15,430 (35.2)	11,158 (41.2)	<0.01
Antihypertension medication, *n* (%)	330 (1.7)	310 (5.4)	3,395 (7.7)	5,426 (20.0)	<0.01

*Data are median (IQR) or n (%) of the group*.

*BMI, body mass index; BP, blood pressure; DBP, diastolic blood pressure; eGFR, estimated glomerular filtration rate; FBG, fasting blood glucose; HDL-C, high-density lipoprotein cholesterol; IQR, interquartile range; LDL-C, low-density lipoprotein cholesterol; No., number; SBP, systolic blood pressure; TC, total cholesterol; TG, triglycerides*.

### Changes in BP and the Occurrence of CVEs

The mean SBP and DBP of the participants at baseline (year 2006–2007) were 131 and 84 mmHg, respectively; however, these BP levels changed to 139 and 83 mmHg after 10 years of follow-up (year 2016–2017), wherein 36,090 new incident hypertension cases were observed. Among the four BP groups defined at baseline, three groups (except BP of ≥140/≥90 mmHg) experienced an increase in mean SBP during follow-up (all *P* < 0.05). Two groups with BP of <120/<80 and 120–129/<80 mmHg also experienced an increase in DBP, while the other two groups exhibited a decreasing trend (both *P* < 0.05) (Supplementary Figure 2). To precisely estimate the association of the different durations of high BP exposure and the occurrence of CVEs, time-weighted cumulative BP determinations between the baseline period and each follow-up interval were calculated. Table 2 shows the distribution of participants in the different time-weighted cumulative BP groups and the corresponding CVEs in each follow-up.

**Table 2:** Risk of occurrence of cardiovascular events estimated based on time-weighted cumulative BP.

**BP categories, mmHg**	**Cases/total**	**Incidence density (per 1,000 person-years)**	**HR (95% CI)**		
			**Model 1**	**Model 2**	**Model 3**
**The whole follow-up period**					
<120/<80	401/17,467	2.16	Reference	Reference	Reference
120–129/<80	291/8,795	3.12	0.94 (0.81–1.10)	0.96 (0.82–1.12)	0.99 (0.85–1.16)
130–139/80–89	2,759/47,564	5.55	1.29 (1.15–1.44)	1.31 (1.17–1.47)	1.34 (1.20–1.51)
≥140/≥90	2,850/21,876	13.30	2.03 (1.80–2.30)	2.06 (1.82–2.33)	2.06 (1.81–2.35)
**The 2nd year since baseline (year 2008–2009)**					
<120/<80	74/20,463	1.81	Reference	Reference	Reference
120–129/<80	38/6,354	3.01	0.86 (0.58–1.27)	0.85 (0.58–1.27)	0.85 (0.57–1.26)
130–139/80–89	368/44,984	4.12	0.90 (0.69–1.18)	0.91 (0.69–1.18)	0.87 (0.67–1.14)
≥140/≥90	479/23,901	10.16	1.13 (0.82–1.55)	1.14 (0.83–1.56)	1.05 (0.76–1.44)
**The 4th year since baseline (year 2010–2011)**					
<120/<80	80/20,119	2.00	Reference	Reference	Reference
120–129/<80	51/6,484	3.97	1.10 (0.78–1.57)	1.10 (0.77–1.57)	1.08 (0.76–1.54)
130–139/80–89	440/45,690	4.86	1.15 (0.89–1.49)	1.17 (0.90–1.51)	1.12 (0.87–1.49)
≥140/≥90	504/21,966	11.69	1.71 (1.26–2.32)	1.73 (1.28–2.35)	1.62 (1.20–2.20)
**The 6th year since baseline (year 2012–2013)**					
<120/<80	74/19,426	1.92	Reference	Reference	Reference
120–129/<80	50/6,555	3.86	1.18 (0.82–1.70)	1.17 (0.82–1.68)	1.15 (0.80–1.65)
130–139/80–89	505/45,843	5.58	1.46 (1.12–1.89)	1.46 (1.13–1.90)	1.39 (1.07–1.81)
≥140/≥90	432/20,305	10.90	1.86 (1.36–2.53)	1.86 (1.37–2.52)	1.73 (1.27–2.35)
**The 8th year since baseline (year 2014–2015)**					
<120/<80	52/18,216	1.44	Reference	Reference	Reference
120–129/<80	58/7,359	3.99	1.76 (1.20–2.56)	1.77 (1.21–2.58)	1.70 (1.16–2.48)
130–139/80–89	518/44,819	5.86	2.02 (1.50–2.74)	2.07 (1.53–2.80)	1.92 (1.42–2.59)
≥140/≥90	492/19,112	13.24	3.00 (2.14–4.20)	3.06 (2.19–4.28)	2.73 (1.96–3.80)
**The 10th year since baseline (year 2016–2017)**					
<120/<80	98/16,663	2.01	Reference	Reference	Reference
120–129/<80	122/8,297	4.98	1.70 (1.30–2.22)	1.69 (1.29–2.21)	1.62 (1.24–2.12)
130–139/80–89	1,015/43,655	7.86	2.16 (1.73–2.68)	2.14 (1.72–2.66)	2.02 (1.62–2.51)
≥140/≥90	851/18,196	16.08	3.11 (2.44–3.96)	3.07 (2.41–3.91)	2.81 (2.21–3.56)

*Model 1: adjusted for age, gender, body mass index, smoking, alcohol consumption, physical activity, systolic blood pressure, and diastolic blood pressure at baseline. Model 2: model 1 plus total cholesterol levels, low-density lipoprotein cholesterol, fasting plasma glucose, and estimated glomerular filtration rate at baseline. Model 3: model 2 plus history of hypertension and antihypertensive drug intake at baseline, variability of systolic blood pressure, and diastolic blood pressure during follow-up*.

*CI, confidence interval; HR, hazard ratio*.

**Figure 1:**
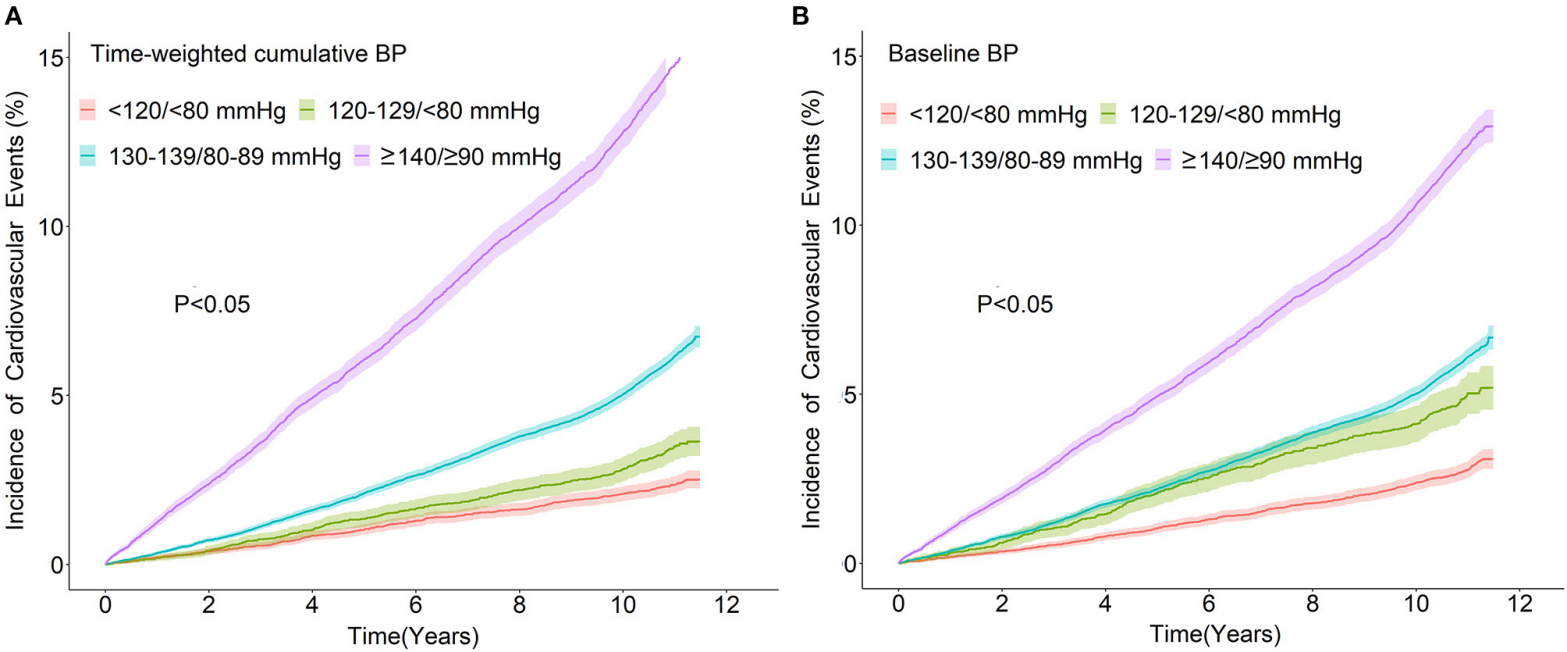
Incidence of cardiovascular events over time according to different categories of time-weighted cumulative BP **(A)** or baseline BP **(B)**. BP, blood pressure.

During the whole follow-up period, we observed 6,301 incident CVEs, including 1,034 with MI, 3,810 with stroke, and 1,457 succumbing to cardiovascular death. The cumulative incident density rates of CVEs during the whole follow-up in the four time-weighted cumulative BP groups—BP <120/<80, 120–129/<80, 130–139/80–89, and ≥140/≥90 mmHg—were 2.16, 3.12, 5.55, and 13.30 per 1,000 person-years, respectively (Table 2). The incidence rates of CVEs during each follow-up according to the time-weighted cumulative BP groups and baseline BP group are shown in Figures 1A,B, respectively. The BP groups with ≥140/≥90 mmHg at baseline BP and time-weighted cumulative BP both exhibited the highest incidence density of CVEs (both *P* < 0.05).

### Difference Between Baseline BP and Time-Weighted Cumulative BP in the Estimation of CVE Risk of Higher BP Exposure

**Figure 2:**
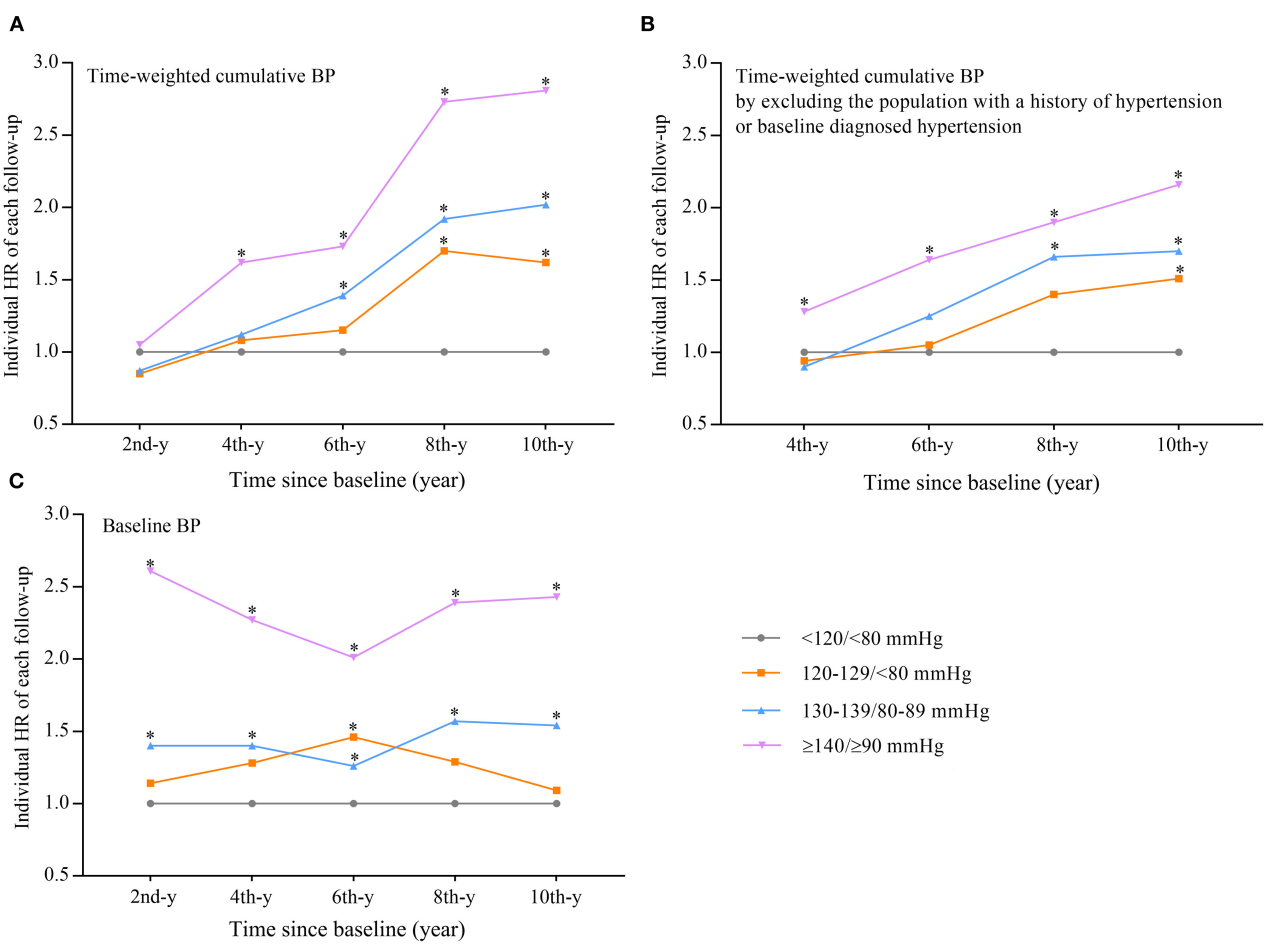
The trend of HR influence of time-weighted cumulative BP **(A)** or time-weighted cumulative BP that excluded the participants with a history of hypertension or baseline diagnosed hypertension **(B)** or baseline BP **(C)** on new occurrence of cardiovascular events during follow-up. **(A,C)** Adjusted for the same variables indicated in model 3 in Table 2 or Supplementary Table 1. **(B)** Adjusted for the same variables indicated in model 3 of Table 2 at the second year since baseline. ^*^Compared with BP of <120/<80 mmHg, the difference was significant (*P* < 0.05). The *x*-y: the *x*-year since baseline. BP, blood pressure; HR, hazard ratio.

To determine the difference between baseline BP and time-weighted cumulative BP in the estimation of CVE risk, we compared their association with the corresponding incident CVEs in each follow-up interval using the multiple variable adjusted Cox regression model. We found a time trend of HR when the estimation was based on the time-weighted cumulative BP. Firstly, HR increased with the increases of higher BP exposure duration, whereby in the BP group of ≥140/≥90 mmHg, the HRs from the first follow-up to the fifth follow-up were 1.05, 1.62, 1.73, 2.73, and 2.81, respectively, compared with the BP group of <120/<80 mmHg. The results from BP groups of 120–129/<80 and 130–139/80–89 mmHg were similar. We further found that the time trend of HR occurred across the different BP groups compared with the time-weighted cumulative BP of <120/<80 mmHg, whereby BP of ≥140/≥90 mmHg exhibited its CVE risk at the second follow-up (the 4th year since baseline) (HR = 1.62, 95% CI 1.20–2.20), BP of 130–139/80–89 mmHg at the fourth follow-up (the 6th year since baseline) (HR = 1.39, 95% CI 1.07–1.81), and a BP of 120–129/<80 mmHg exhibited unfavorable effects on CVEs at the sixth follow-up (the 8th year since baseline) (HR = 1.70, 95% CI 1.96–3.80) (Table 2). The trends of HR for each time-weighted cumulative BP group during these follow-up intervals are summarized in Figure 2A.

**Figure 3:**
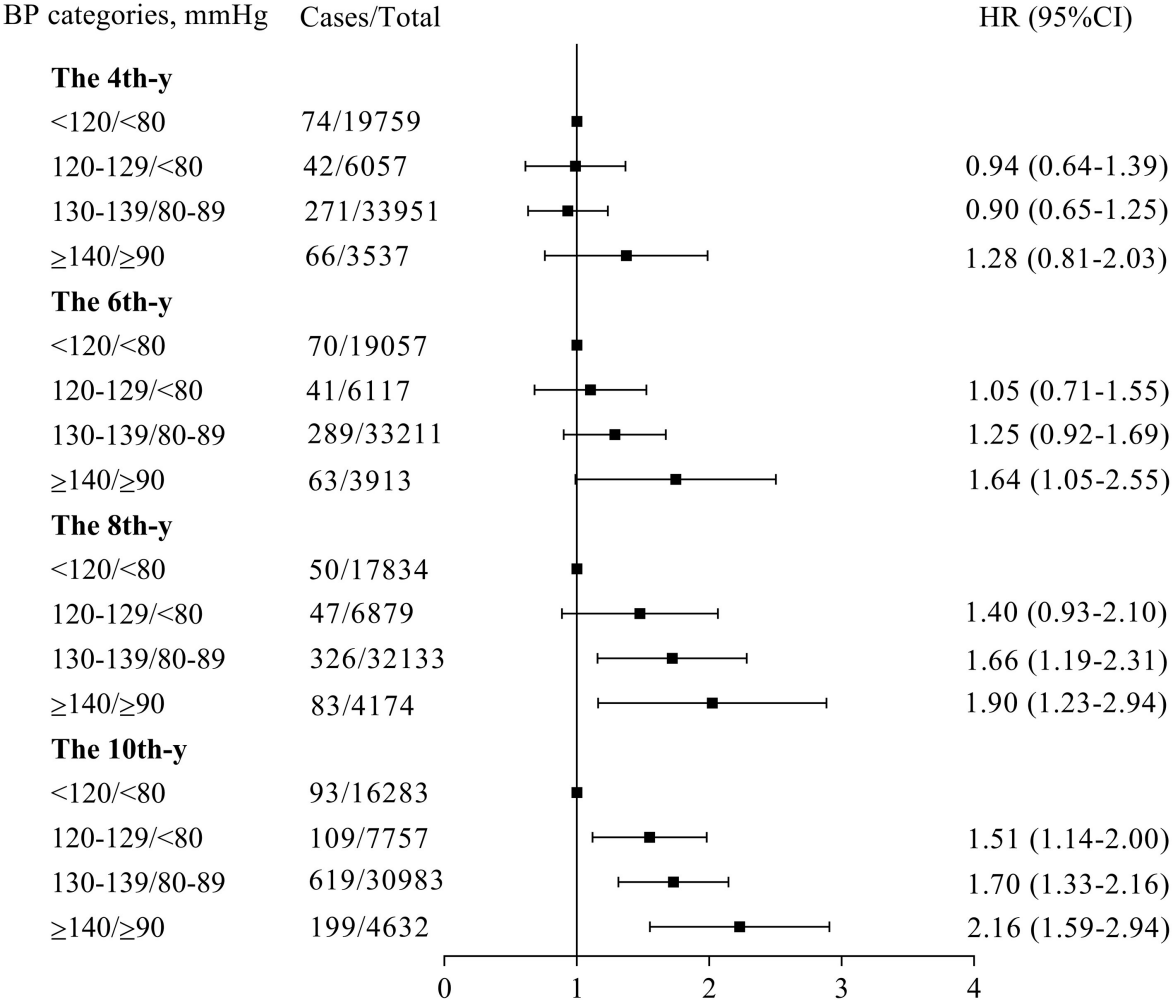
Risks of new occurrence cardiovascular events in participants with different categories of time-weighted cumulative BP after excluding the participants with a history of hypertension or baseline diagnosed hypertension. This analysis was adjusted for the same variables indicated in Figure 2B. ^*^The *x*-y: the *x*-year since baseline. BP, blood pressure; CI, confidence interval; HR, hazard ratio.

To eliminate the influence of higher BP on CVE occurrence before baseline, the same analysis was performed on the participants without baseline hypertension or a history of hypertension; the results were similar to the analysis performed on the whole participant population (Figure 3), and the trends of HRs over time are summarized in Figure 2B. However, when the estimation was performed based on baseline BP, the time trend observed with cumulative BP would no longer exist (Supplementary Table 1, Figure 2C).

We had also performed a multivariable adjusted restricted spline regression model to examine what was the most favorable level of BP at which participants had the lowest risk of CVE occurrence. We found a J-shaped association between time-weighted cumulative SBP (Supplementary Figure 3A) or DBP (Supplementary Figure 3B) and the risk of CVE occurrence, which demonstrated that the time-weighted cumulative SBP at 122 mmHg (range: 121–126 mmHg) and DBP at 78 mmHg (range: 76–80 mmHg), respectively, had the lowest risk of CVE occurrence.

### The Predictive Value of Baseline BP and Time-Weighted Cumulative BP Determinations for the Incidence of CVEs

Since baseline BP is commonly used in the prediction of CVE occurrence currently, we examined the extent that baseline BP alone could precisely predict the occurrence of CVEs by performing a ROC analysis. We compared the AUCs from the model that considered only the baseline BP with the “combined” model that considered both baseline BP and time-weighted cumulative BP determined at each follow-up. We found that although the combined model significantly improved the predictive ability compared with the baseline BP alone model (all *P* < 0.05) (Supplementary Figures 4, 5), the predictive ability (AUC) was increased only by 0.6–3.2% for SBP (Supplementary Figure 4F) and 0.2–3.1% for DBP (Supplementary Figure 5F), respectively, during the five intervals of follow-up.

## Discussion

In this prospective study, we had examined the difference between baseline BP and time-weighted cumulative BP determinations in the estimation of the effect of higher BP exposure on CVE occurrence. We demonstrated that when HR was estimated based on time-weighted cumulative BP determination, a reasonable time trend of HRs was clearly shown both in the same BP group and across the four BP groups; these results were confirmed by the same analysis performed on participants without baseline hypertension. However, these time trends did not exist when the estimation was based on baseline BP determination. We also demonstrated that the predictive value of baseline BP determination for the occurrence of CVEs was only slightly lower (<4%) compared with the time-weighted cumulative BP determination.

The current estimation of the effect of BP on CVE occurrence is mainly based on a single baseline BP measurement. However, there is a gap in time between baseline BP determination and risk estimation. Intuitively, this influence on CVEs would accumulate with a sustained duration of high BP exposure, and a time-weighted cumulative BP strategy was employed to assess the risk of CVE in this study. Indeed, we found that the HR of higher BP increased with the increase in duration of the higher BP (Table 2, Figure 2A), and this time trend also occurred across the four time-weighted BP groups, with the BP group of ≥140/≥90 mmHg being the earliest to exhibit its CVE risk, followed by BP groups of 130–139/80–89 and 120–129/<80 mmHg (Table 2, Figure 2A).

Although we had adjusted the baseline BP in the above analysis, to more precisely assess the influence of the different durations of high BP exposure on CVE occurrence, the influence of high BP before baseline should be completely eliminated. Therefore, we performed the same analysis on the participants without baseline hypertension, and the results were similar as the above analysis (Figures 2B, 3). However, these time trends no longer existed when the time-weighted BP determination was substituted with baseline BP determination (Supplementary Table 1, Figure 2C). Currently, due to the lack of clear objective criteria, such as C-statistics or NRI in evaluating the improvement of predictive value (15, 16), to directly compare the superiority of baseline BP and cumulative BP determination in risk estimation, however, based on time-weighted cumulative BP determination, could more accurately assess the extent of BP exposure theoretically and could have a reasonable time trend of HR of risk estimation in the population. We conclude that the risk of higher BP on CVE occurrence estimated based on time-weighted BP determination could be more precise than that of baseline BP determination.

Although cumulative BP determination is shown in this study to be a better estimation of the risk of high BP on CVE occurrence, the greater accessibility and reduced resource utilization in performing baseline BP determination are good reasons to continue using this to predict the risk of CVE occurrence; therefore, further assessment of its accuracy needs to be conducted. We compared the predictive values (AUC) of the baseline BP for CVE occurrence with the combined model of baseline BP plus time-weighted cumulative BP and found that the difference was statistically significant. This result was similar to the Lifetime Risk Pooling Project cohorts study (16). However, the AUC from the model that included baseline BP alone was only slightly lower (<4%) than that from the combined model (Supplementary Figures 4, 5), which suggests that baseline BP might still be a useful indicator for predicting the future occurrence of CVEs.

To assess for the optimal range of BP in which participants would be at relatively lower risk of the occurrence of CVEs, we performed a restricted cubic spline regression analysis and found that SBP at 122 mmHg and DBP at 78 mmHg exhibited the lowest risk for CVE occurrence, with their corresponding ranges being 121–126 and 76–80 mmHg, respectively. This result indicated that participants would benefit from the BP maintained at these ranges in reducing their risks for CVEs.

This study has several strengths. First, the data of this analysis were derived from a large-scale, community-based prospective study. Second, we had used the time-weighted cumulative BP determination to measure the extent of BP exposure since baseline; thus, both BP level and the corresponding duration of exposure were considered. Third, the results were confirmed in participants without baseline hypertension, which would eliminate the influence of hypertension before baseline, thus providing a precise estimate of the different durations of high BP on CVE occurrence. Fourth, since BP level varies over time, the duration of such BP levels would be expected to have an influence on CVE occurrence (29, 30); thus, in this study, we adjusted the variation of BP level from five times of follow-up. In addition, we had also adjusted for other potential confounding factors, such as death competition risk to CVEs and history of antihypertension drug used.

There were also several limitations of this study. First, we used the cumulative BP exposure for each follow-up to estimate the corresponding risk, which poses an intrinsic limitation; such an estimation means we considered that high BP has the same influence on CVE occurrence in different stages of life: in elderly people, high BP may not be so significant as in relatively young people (4, 31). Second, in routine clinical practice, only baseline BP makes it possible to estimate CVE risk (such as the Framingham score), and that it remains necessary to develop risk estimators whose estimate of the risk evolves over time by taking into account the changes of different parameters. Third, considering the great advances in the management of hypertension over the past decade, the generalizability of the conclusion may be limited.

## Conclusions

In this study, we first confirmed that baseline BP is a reliable indicator for predicting the occurrence of CVEs. Nevertheless, time-weighted cumulative BP could be a more reliable estimate of the risk of CVEs than baseline BP.

## Data Availability Statement

The raw data supporting the conclusions of this article will be made available by the authors, without undue reservation.

## Ethics Statement

The studies involving human participants were reviewed and approved by The study was performed according to the guidelines of the Helsinki Declaration and was approved by the Ethics Committee of Kailuan General Hospital (approval number: 2006-05) and Beijing Tiantan Hospital (approval number: 2010-014-01). The patients/participants provided their written informed consent to participate in this study.

## Author Contributions

SW and YH: had full access to all the data in the study and take responsibility for the integrity of the data and the accuracy of the data analysis. YZu and AW: concept and design. DZ, YY, XY, and FL: acquisition, analysis, or interpretation of data. YZu and AW: drafting of the manuscript. YH: critical revision of the manuscript for important intellectual content. SC, XT, MW, XS, JW, QZ, and YZh: statistical analysis. YH: obtained funding. SW and YH: supervision. HG: Other. All authors contributed to the article and approved the submitted version.

## Funding

This study was funded by the National Natural Science Foundation of China (82073648 and 31672375), Beijing Municipal Administration of Hospitals Incubating Program (PX2020021), Beijing Excellent Talents Training Program (2018000021469G234), Young Elite Scientists Sponsorship Program by CAST (2018QNRC001), and National Key R&D Program of China (2018YFC1312400 and 2018YFC1312402).

## Conflict of Interest

The authors declare that the research was conducted in the absence of any commercial or financial relationships that could be construed as a potential conflict of interest.

## Publisher's Note

All claims expressed in this article are solely those of the authors and do not necessarily represent those of their affiliated organizations, or those of the publisher, the editors and the reviewers. Any product that may be evaluated in this article, or claim that may be made by its manufacturer, is not guaranteed or endorsed by the publisher.
