# Supplementary material for: Baseline and Cumulative Blood Pressure in Predicting the Occurrence of Cardiovascular Events
Source: Front Cardiovasc Med. 2021 Sep 21;8:735679. doi: 10.3389/fcvm.2021.735679 (PMC8490882; doi:10.3389/fcvm.2021.735679)
Supplement: Supplementary file 1 [file Data_Sheet_1.docx]

**Supplementary Material**

**eTable 1.** Risk of occurrence of cardiovascular events estimated based on baseline BP

**eTable 2.** Antihypertension medications information at each follow-up period of participants according to the baseline BP categories

**eFigure 1.** Flowchart of the study cohort.

**eFigure 2.** Changes of systolic (A) and diastolic BP (B) during follow-up according to BP groups defined at baseline.

**eFigure 3.** HR and 95% confidence intervals for cardiovascular events derived from restricted cubic spline regression, with knots placed at the 5th, 25th, 50th, 75th, and 95th percentiles of the distribution of time-weighted cumulative systolic and diastolic BP.

**eFigure 4.** ROC analysis for baseline SBP and time-weighted cumulative SBP distinguishing new CVE occurrence.

**eFigure 5.** ROC analysis for baseline DBP and time-weighted cumulative DBP distinguishing new CVE occurrence.

eTable 1. Risk of occurrence of cardiovascular events estimated based on baseline BP

| BP categories, mmHg | Cases/Total | Incidence density  (per 1,000 person-years) | HR (95% CI) | | |
| --- | --- | --- | --- | --- | --- |
|  |  |  | Model 1 | Model 2 | Model 3 |
| The whole follow-up period |  |  |  |  |  |
| <120/<80 | 515/18999 | 2.55 | Reference | Reference | Reference |
| 120-129/<80 | 269/5781 | 4.46 | 1.32 (1.14-1.53) | 1.29 (1.11-1.51) | 1.22 (1.04-1.42) |
| 130-139/80-89 | 2513/43847 | 5.50 | 1.61 (1.46-1.77) | 1.59 (1.44-1.75) | 1.42 (1.28-1.56) |
| ≥140/≥90 | 3004/27075 | 11.12 | 2.86 (2.60-3.14) | 2.82 (2.56-3.11) | 2.25 (2.03-2.50) |
| The 2nd-y since baseline (year 2008-2009) |  |  |  |  |  |
| <120/<80 | 67/18999 | 1.77 | Reference | Reference | Reference |
| 120-129/<80 | 35/5781 | 3.04 | 1.22 (0.81-1.84) | 1.22 (0.81-1.84) | 1.14 (0.76-1.72) |
| 130-139/80-89 | 343/43847 | 3.93 | 1.58 (1.22-2.06) | 1.59 (1.22-2.07) | 1.40 (1.07-1.82) |
| ≥140/≥90 | 514/27075 | 9.62 | 3.37 (2.60-4.36) | 3.38 (2.61-4.38) | 2.61 (2.00-3.40) |
| The 4th-y since baseline (year 2010-2011) |  |  |  |  |  |
| <120/<80 | 83/18871 | 2.21 | Reference | Reference | Reference |
| 120-129/<80 | 48/5716 | 4.24 | 1.37 (0.96-1.96) | 1.38 (0.96-1.97) | 1.28 (0.90-1.83) |
| 130-139/80-89 | 417/43304 | 4.86 | 1.56 (1.23-1.98) | 1.59 (1.25-2.02) | 1.40 (1.10-1.78) |
| ≥140/≥90 | 527/26368 | 10.16 | 2.85 (2.25-3.62) | 2.93 (2.30-3.72) | 2.27 (1.77-2.91) |
| The 6th-y since baseline (year 2012-2013) |  |  |  |  |  |
| <120/<80 | 94/18668 | 2.53 | Reference | Reference | Reference |
| 120-129/<80 | 60/5607 | 5.43 | 1.58 (1.14-2.19) | 1.57 (1.13-2.17) | 1.46 (1.06-2.02) |
| 130-139/80-89 | 410/42439 | 4.89 | 1.41 (1.12-1.76) | 1.42 (1.14-1.78) | 1.26 (1.00-1.58) |
| ≥140/≥90 | 497/25415 | 10.00 | 2.53 (2.02-3.16) | 2.55 (2.04-3.19) | 2.01 (1.59-2.54) |
| The 8th-y since baseline (year 2014-2015) |  |  |  |  |  |
| <120/<80 | 87/18412 | 2.38 | Reference | Reference | Reference |
| 120-129/<80 | 47/5451 | 4.36 | 1.40 (0.98-2.01) | 1.40 (0.98-2.00) | 1.29 (0.90-1.85) |
| 130-139/80-89 | 462/41345 | 5.67 | 1.77 (1.41-2.24) | 1.79 (1.42-2.26) | 1.57 (1.24-1.98) |
| ≥140/≥90 | 524/24298 | 11.05 | 3.08 (2.44-3.88) | 3.11 (2.46-3.93) | 2.39 (1.87-3.04) |
| The 10th-y since baseline (year 2016-2017) |  |  |  |  |  |
| <120/<80 | 184/18165 | 3.46 | Reference | Reference | Reference |
| 120-129/<80 | 79/5317 | 5.05 | 1.14 (0.88-1.49) | 1.14 (0.87-1.48) | 1.09 (0.84-1.42) |
| 130-139/80-89 | 881/40161 | 7.42 | 1.65 (1.41-1.94) | 1.66 (1.41-1.95) | 1.54 (1.31-1.82) |
| ≥140/≥90 | 942/23168 | 13.93 | 2.77 (2.36-3.26) | 2.79 (2.37-3.28) | 2.43 (2.05-2.88) |

Abbreviations: CI, confidence interval; HR, hazard ratio.

Model 1: Adjusted for age, gender, body mass index, smoking, alcohol consumption, , physical activity at baseline. Model 2: Model 1 plus total cholesterol levels, low-density lipoprotein cholesterol, fasting plasma glucose and estimated glomerular filtration rate at baseline. Model 3: Model 2 plus history of hypertension and antihypertensive drug intake at baseline.

eTable 2. Information on antihypertensive medications at each follow-up period of participants according to the baseline BP categories

|  | <120/<80 mmHg | 120-129/<80 mmHg | 130-139/80-89 mmHg | ≥140/≥90 mmHg |
| --- | --- | --- | --- | --- |
| Antihypertensive medication, n (%) |  |  |  |  |
| Baseline (year 2006-2007) | 330 (1.7) | 310 (5.4) | 3395 (7.7) | 5426 (20.0) |
| The 2nd-y since baseline (year 2008-2009) | 234 (1.6) | 191 (4.4) | 2147 (6.8) | 3787 (21.7) |
| The 4th-y since baseline (year 2010-2011) | 335 (2.4) | 259 (6.5) | 2821 (9.4) | 3899 (23.3) |
| The 6th-y since baseline (year 2012-2013) | 371 (2.7) | 290 (7.4) | 2831 (9.7) | 3734 (22.2) |
| The 10th-y since baseline (year 2016-2017) | 266 (2.7) | 183 (6.9) | 2065 (10.3) | 2415 (21.8) |

The antihypertensive medication information in period of year 2014-2015 was not collected.


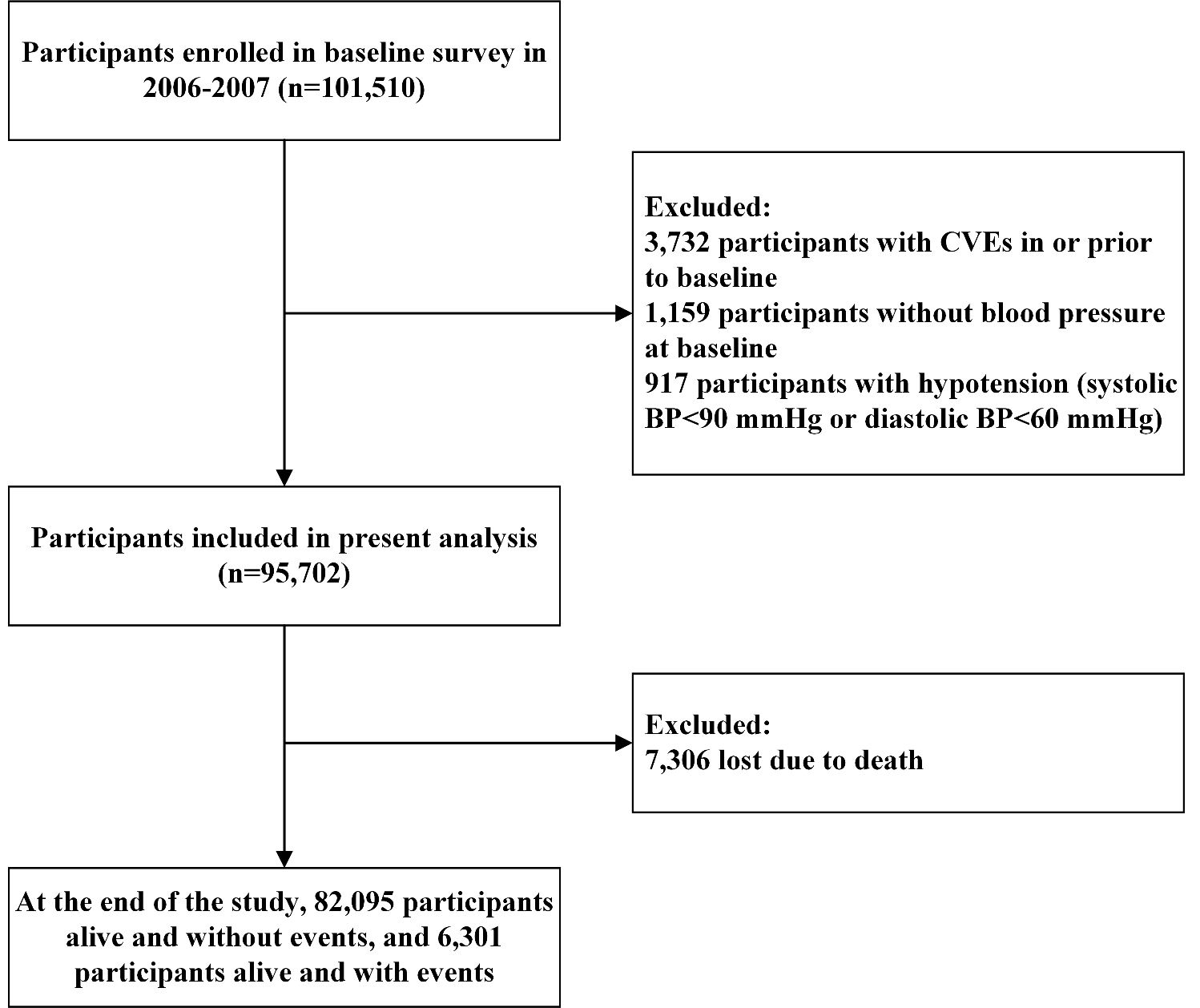


eFigure 1. Flowchart of the study cohort. Abbreviations: BP, blood pressure; CVEs, cardiovascular events


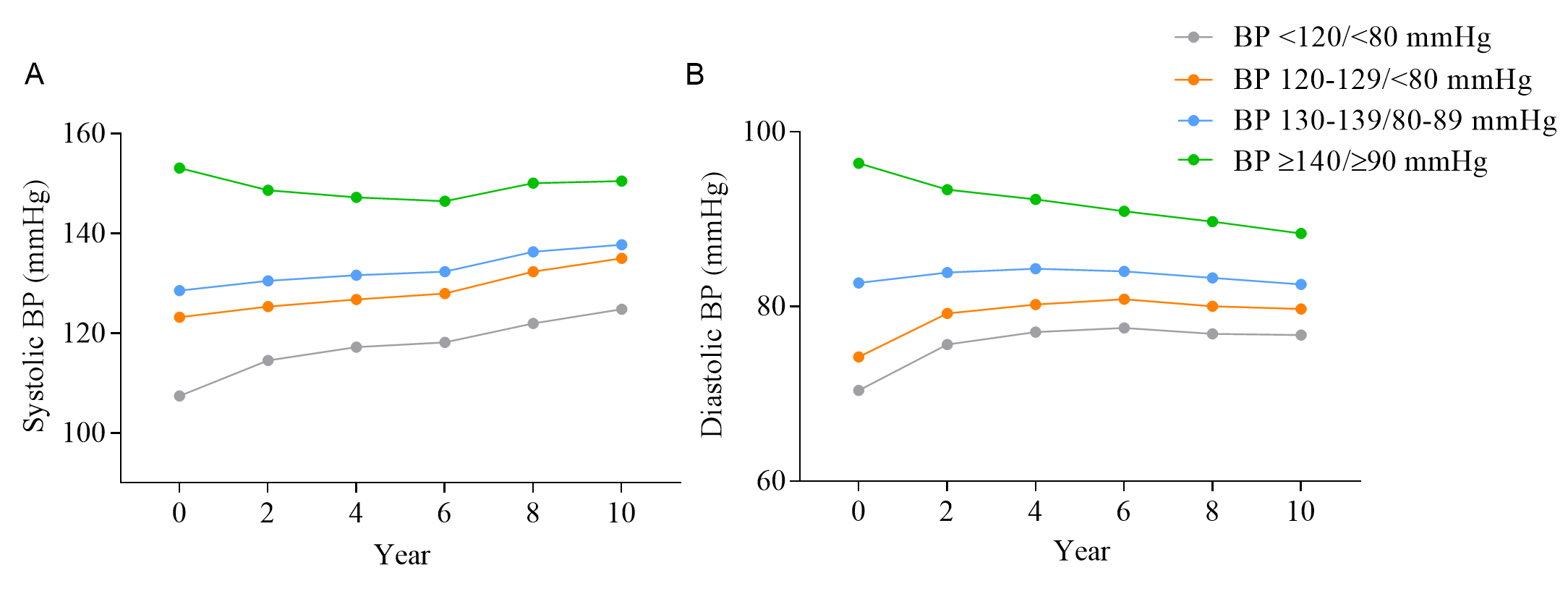


eFigure 2. Changes of systolic (A) and diastolic BP (B) during follow-up according to BP groups defined at baseline. Abbreviations: BP, blood pressure


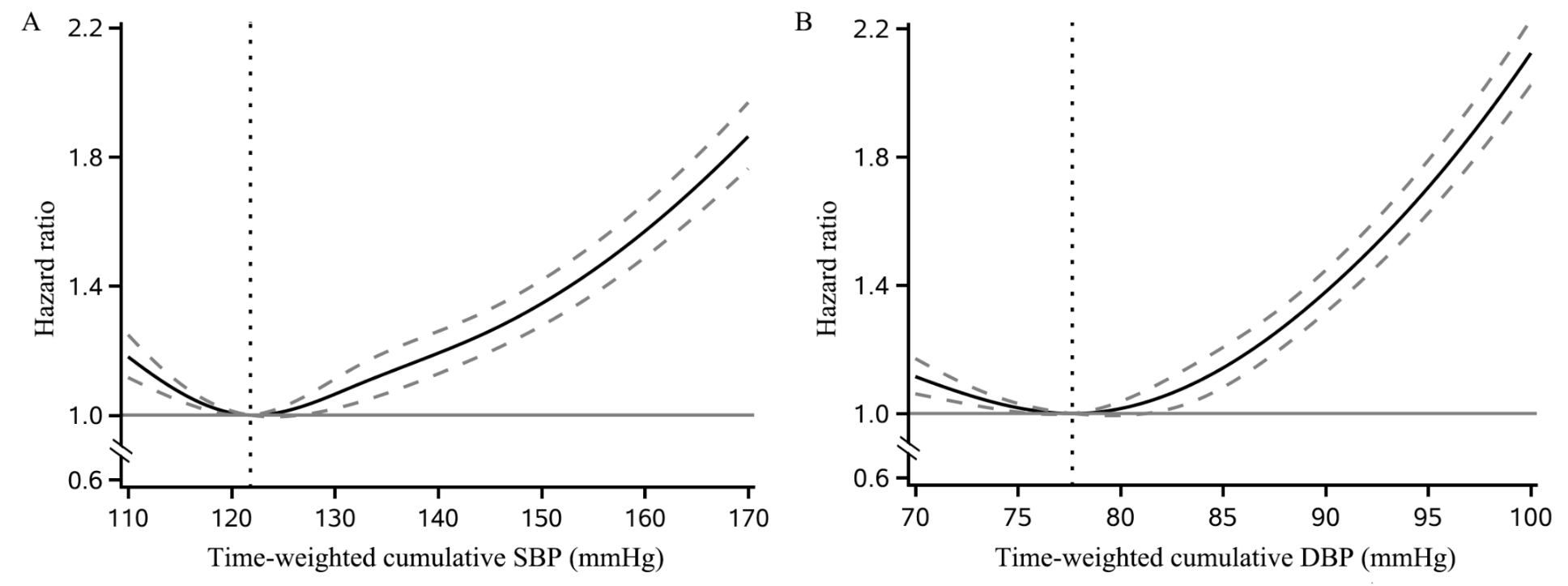


eFigure 3. HR and 95% confidence intervals for cardiovascular events derived from restricted cubic spline regression, with knots placed at the 5th, 25th, 50th, 75th, and 95th percentiles of the distribution of time-weighted cumulative systolic and diastolic BP. This analysis was adjusted for the same variables indicated in the model 3 in Table 3. Systolic (A) and diastolic BP (B). Abbreviations: BP, blood pressure; HR, hazard ratio.


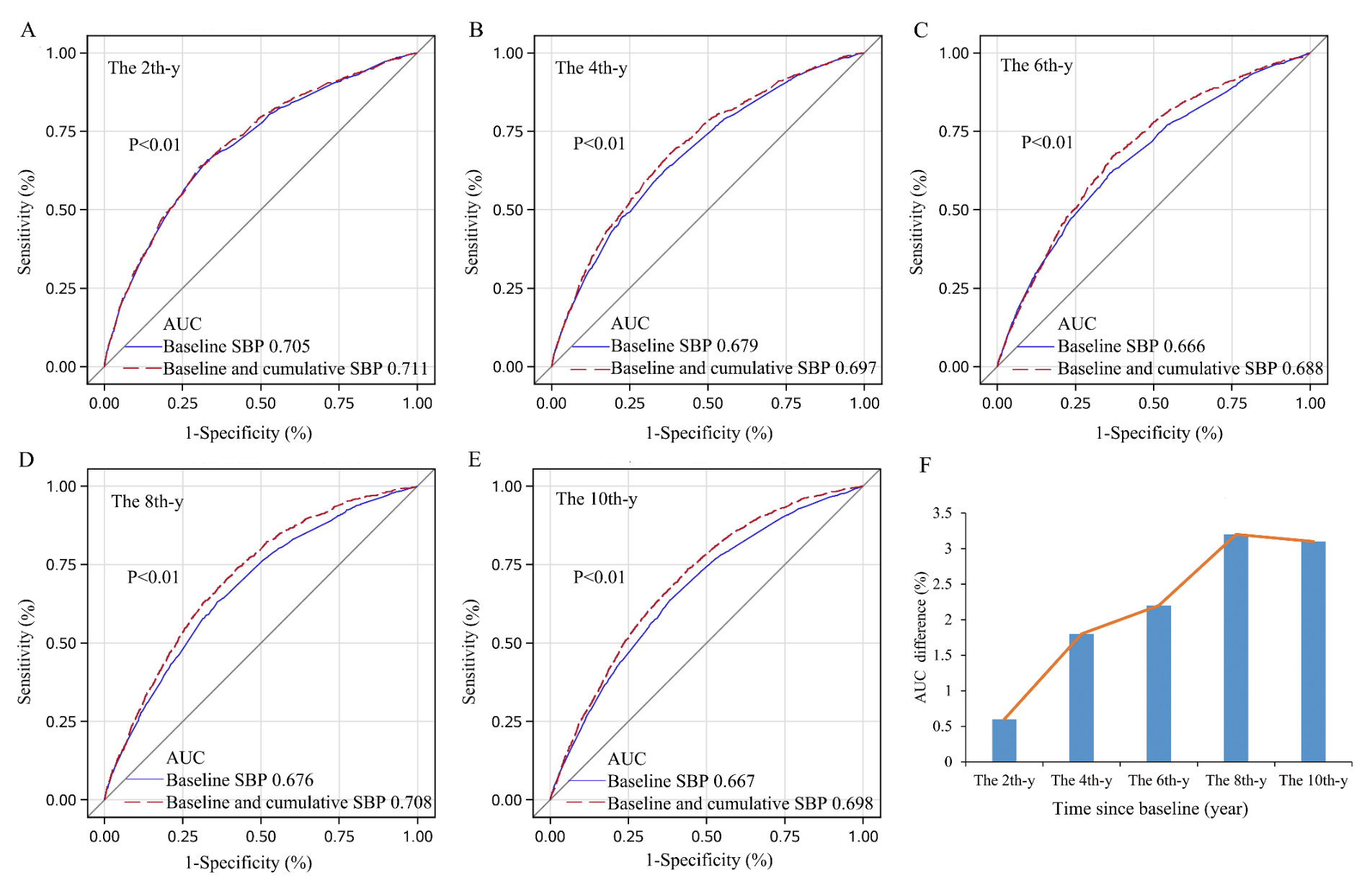


eFigure 4. ROC analysis for baseline SBP and time-weighted cumulative SBP distinguishing new CVE occurrence. (F) The predictive value (AUC) difference between baseline SBP and time-weighted cumulative SBP for CVEs in the different time periods. Abbreviations: AUC, area under curve; CVEs, cardiovascular events; ROC, receiver operating characteristic; SBP, systolic blood pressure.


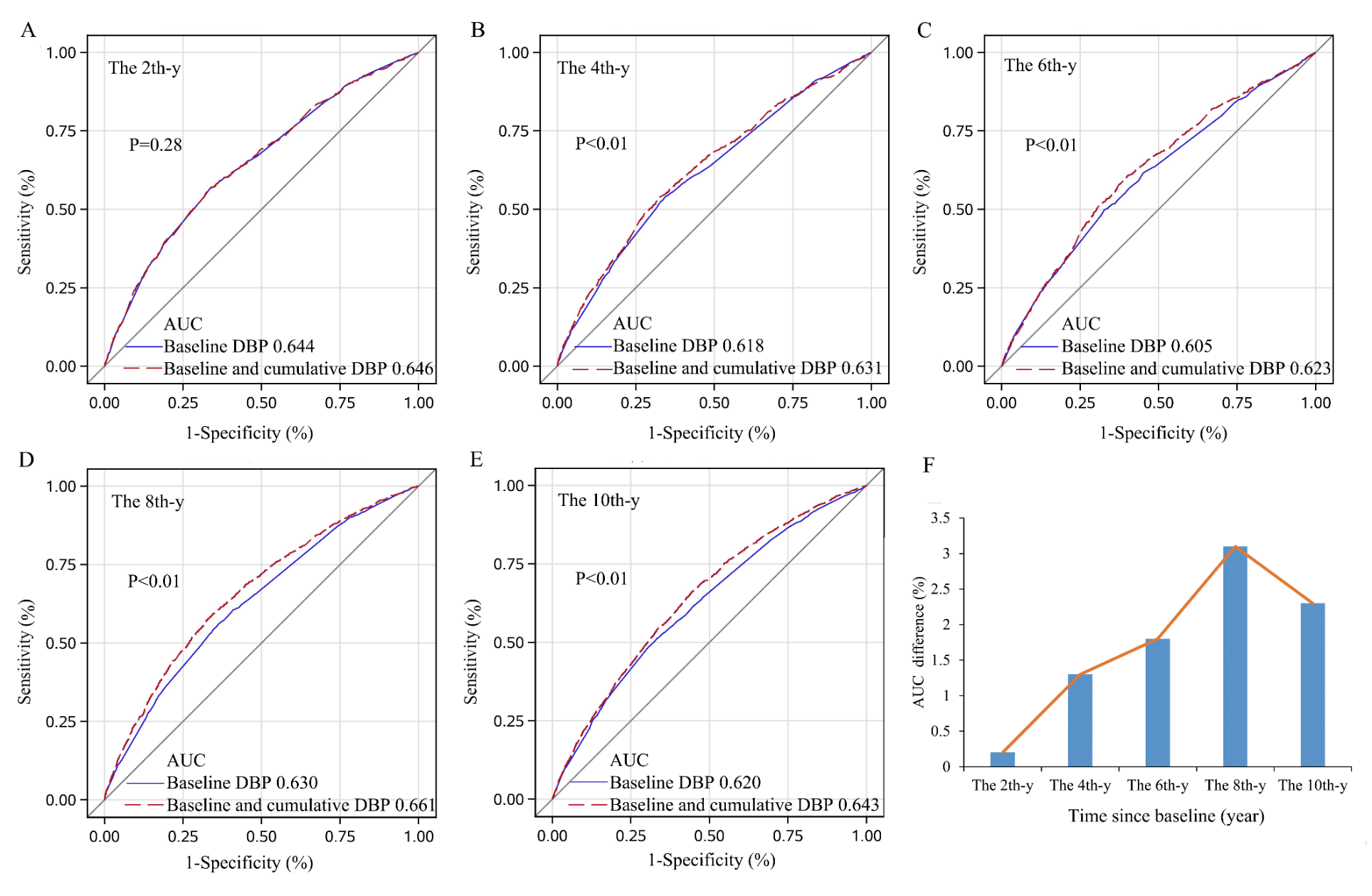


eFigure 5. ROC analysis for baseline DBP and time-weighted cumulative DBP distinguishing new CVE occurrence. (F) The predictive value (AUC) difference between baseline DBP and time-weighted cumulative DBP for CVEs in the different time periods. Abbreviations: AUC, area under curve; CVEs, cardiovascular events; DBP, diastolic blood pressure; ROC, receiver operating characteristic.
